# Audiometric findings with voluntary tensor tympani contraction

**DOI:** 10.1186/s40463-016-0182-y

**Published:** 2017-01-05

**Authors:** Brandon Wickens, Duncan Floyd, Manohar Bance

**Affiliations:** 1 Division of Otolaryngology-Head and Neck Surgery, McMaster University, Hamilton, ON Canada; 2 School of Human Communication Disorders, Dalhousie University, Hamilton, NS Canada; 3 Division of Otolaryngology, QEII Health Sciences Centre, Dalhousie University, Room 3184, 3rd Floor Dickson Building, VG site, 1278 Tower Road, Hamilton, NS B3H 2Y9 Canada

**Keywords:** Tensor tympani, Audiometry, Audiology, Middle ear

## Abstract

**Background:**

Tensor tympani contraction may have a "signature" audiogram. This study demonstrates audiometric findings during voluntary tensor tympani contraction.

**Methods:**

Five volunteers possessing the ability to voluntarily contract their tensor tympani muscles were identified and enrolled. Tensor tympani contraction was confirmed with characteristic tympanometry findings. Study subjects underwent conventional audiometry. Air conduction and bone conduction threshold testing was performed with and without voluntary tensor tympani contraction.

**Main outcome measure:**

Changes in air conduction and bone conduction thresholds during voluntary tensor tympani contraction.

**Results:**

Audiometric results demonstrate a low frequency mixed hearing loss resulting from tensor tympani contraction. Specifically, at 250 Hz, air conduction thresholds increased by 22 dB and bone conduction thresholds increased by 10 dB.

**Conclusions:**

Previous research has demonstrated a low frequency conductive hearing loss in the setting of tensor tympanic contraction. This is the first study to demonstrate a low frequency mixed hearing loss associated with tensor tympani contraction. This finding may aid in the diagnosis of disorders resulting from abnormal tensor tympani function. Tensor tympani contraction should be included on the differential for low frequency mixed hearing loss.

## Background

The contribution of the tensor tympani muscle to middle ear function and dysfunction remains a topic of controversy. EMG studies have demonstrated TT activity during vocalization, yawning, swallowing, laughing, coughing, and both face and head movements, but not to auditory stimuli [1, 2]. It has been proposed that the TT contributes to eustachian tube function and even to high frequency sound transmission by impedance modulation [3]. Additionally, authors have implicated TT dysfunction in several otologic symptoms and conditions, including tinnitus [4], aural fullness, eustachian tube dysfunction [5] and Meniere’s disease [6]. Several authors have proposed surgeries of the TT as plausible treatment options for multiple otologic conditions [4, 6–10].

In order to better understand the role of the TT in otologic symptoms and conditions, it is important to establish objective indicators of TT function. Recently, our group (Aron et al [11]) identified several markers of tensor tympani contraction using voluntary eardrum movement and temporal bone experiments as models for TT contraction. These markers are: low static middle ear compliance, asymmetry in the tympanometry curve during a pressure sweep from positive to negative pressure, and negative measured middle ear pressure. Most specific to TT contraction, and a clear differentiator from stapedial contraction, was the reversal of the positive deflection during tympanometry testing. A positive deflection at ambient external canal pressure reversed to become a negative deflection at negative external canal pressure in the setting of TT contraction.

Determining the audiometric effects of TT contraction may provide additional objective evidence in the form of characteristic audiograms thereby implicating its possible contribution to otologic conditions. If these audiometric patterns are seen, then the tympanometric markers could also be sought. Additionally, an accurate audiometric characterization may allow the implication of TT dysfunction in cases of hearing loss, or other otologic symptoms. Politzer articulated the phenomenon of TT contraction and its association with hearing loss in his 1909 textbook, where he described “a deafness that occurs during yawning brought on by simultaneous contraction of the tensor tympani” [12]. Although case reports in the literature have documented the audiometric effect of TT contraction [13–15], these examples identified a low frequency conductive hearing loss associated with voluntary TT contraction.

Our group (Bance et al, [16]) recently demonstrated a low frequency mixed hearing loss in an individual capable of voluntary TT contraction. We hypothesize that TT contraction results in a low frequency mixed hearing loss rather than purely conductive loss as has previously been demonstrated. To test this hypothesis, individuals capable of voluntary TT contraction were recruited to undergo comprehensive audiometric testing.

## Methods

This study was approved by our institutional research ethics board.

**Fig. 1:**
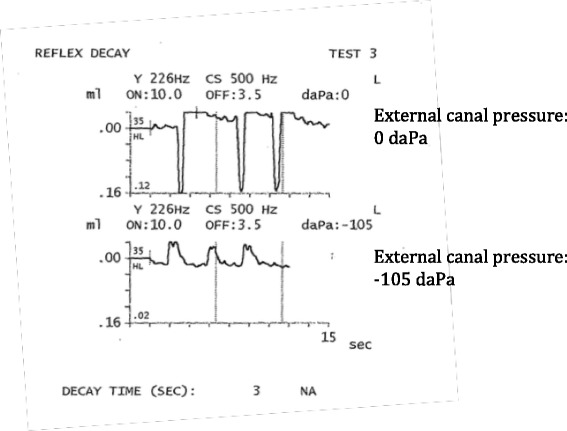
Modified reflex decay test findings used to identify voluntary TT contraction in the test subjects. Here, inversion of the waveform occurs when negative pressure is applied to the external canal during TT contraction

Five subjects capable of voluntary TT contraction were recruited for audiometric testing. TT contraction was confirmed with modified reflex decay testing (Fig. 1), according to the criteria defined by Aron et al [11], and outlined above already. Particularly, all subjects showed compliance change with contraction reverses from ambient or positive pressure to negative pressure in the external ear canal. All subjects met this criterion. Subjects were able to hold contractions long enough to test one frequency at a time. Several subjects fatigued after one ear was tested, and were unable to complete testing of the second side. Consequently, audiometric results were used from only one ear of each participant. Masked bone conduction and air conduction thresholds were tested in all subjects. Pure tone audiograms were collected, analyzed and averaged. Some subjects could not hold their TT contractions long enough to perform air conduction and bone conduction testing in the same session, as contractions seem to become fatigued with time. Hence, some air and bone conduction sessions were performed at different times, or the non-contracted and contracted TT audiograms were performed at different times.

## Results

**Fig. 2:**
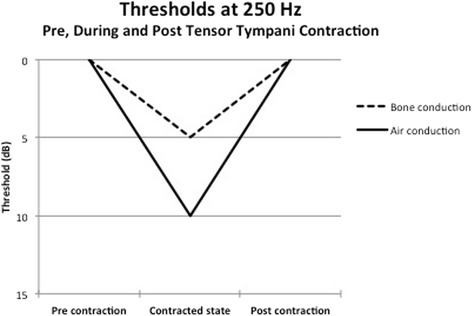
Thresholds in 1 subject at 250 Hz prior to, during, and after tensor tympanic contraction, demonstrating the reversible nature of the mixed hearing loss seen in these subjects capable of voluntary tensor tympani contraction

**Fig. 3:**
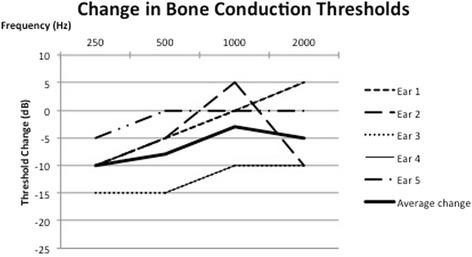
Change in bone conduction thresholds

**Fig. 4:**
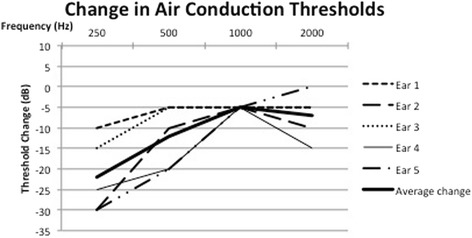
Change in air conduction thresholds

**Fig. 5:**
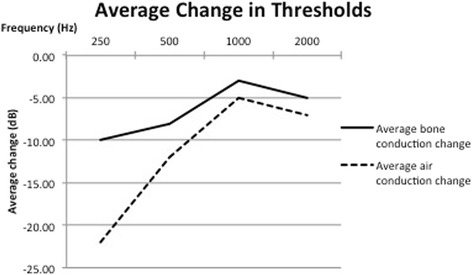
Average change in thresholds

Pure tone audiometry in five subjects with the TT in the relaxed and contracted state was collected. All subjects had normal pre-test air conduction thresholds of less than 25 dB with no pre-existing air bone gap at the tested frequencies. TT contraction produced a mixed hearing loss, with a reduction in air conduction and bone conduction thresholds at 250 Hz, 500 H, 1000 Hz and 2000 Hz during TT contraction. These changes were temporary and reversed with cessation of tensor tympanic contraction. Fig. 2 shows an example of the reversible changes in bone conduction and air conduction thresholds at 250 Hz in one of the subjects. The changes in bone conduction thresholds from baseline are demonstrated in Fig. 3. The changes in air conduction thresholds from baseline are demonstrated in Fig. 4. Average threshold changes are demonstrated in Fig. 5.

## Discussion

The TT muscle measures 20 to 25 mm in length, arises from the cartilaginous Eustachian tube, adjacent sphenoid bone and its bony semi canal; turning on the cochleariform process, it terminates as a tendon inserting on the medial aspect of the malleus [14]. Activation of the TT muscle by the mandibular division of cranial nerve V results in medial displacement of the manubrium and tensing of the tympanic membrane.

The audiometric effect of voluntary TT contraction has been documented previously. In 1943, the case of a lieutenant capable of voluntary TT contraction was reviewed. A low frequency hearing loss was identified during voluntary TT contraction. However, bone conduction thresholds were not tested [13]. In 1960, Reger et al. performed audiometric measurements on eight ears with maximal contraction of the middle ear muscles. Hearing levels were averaged, and a low frequency hearing loss due in the contracted state was identified. Again, bone conduction thresholds were not assessed [17]. In 2013, Angeli et al. describe the case of a 27 year old male presenting with voluntarily evoked bilateral tinnitus. Otoscopy revealed medial displacement of the malleus and tympanic membrane during episodes, consistent with tensor tympani contraction. Bone conduction and air conduction audiometry, conducted during episodes of voluntary TT relaxation and contraction, revealed a low frequency conductive hearing loss [15].

Our group (Pennings et al., [18]) used laser doppler vibrometry to show a reduction in movement of the stapes footplate and tympanic membrane at low frequencies during mass loading of the TT, although it could not be determined whether these changes would result in a conductive or sensorineural hearing loss. Pau et al. discuss the case of an individual who developed recurrent episodes of a 25 dB low frequency sensorineural hearing loss associated with aural fullness, and propose that similar cases of episodic low frequency hearing loss could be attributed to TT dysfunction [19].

In this study, all patients showed varying degrees of reversible low frequency mixed hearing loss during voluntary tensor tympani contraction – a finding that has not been shown previously. The mechanism for this loss is proposed to be due to a combination of effects. TT contraction results in stiffening of the ossicular mechanism, which could result in a conductive hearing loss. The low frequency sensorineural component of hearing loss induced by TT contraction may be theoretically explained by a number of mechanisms. One is the loss of the osteo-tympanal contribution to bone conduction thresholds [19]. In this mechanism, sounds are transmitted by bone conduction to the ossicles, resulting in transmission through the oval window. This phenomenon is proposed to predominate at low frequencies, and loss of this contribution due to TT contraction may result in a low frequency sensorineural hearing loss. Another possible mechanism is a change in the cochlear impedance load seen by bone conduction, caused by medialization of the stapes footplate into the oval window and stretching of the annular ligament and round window membrane. Additionally, TT contraction produces a low frequency noise, described by patients as a low pitched roar [13], and this may result in a masking effect, which is another explanation for the low frequency sensorineural loss seen in this series of patients.

Aaron et al. found that the reversal of the positive deflection on modified reflex decay testing is specific to tensor tympanic contraction [11]. However, this test is only useful in the setting of dynamic, not chronic, tensor tympani contraction. The primary known indicators of chronic tensor tympani contraction are low static middle ear compliance and negative middle ear pressure. This new finding of a mixed low frequency sensorineural hearing loss is important in that could provide an additional indicator of chronic tensor tympanic contraction.

## Conclusion

Our study is the first to demonstrate a low frequency mixed hearing loss resulting from voluntary contraction of the tensor tympani muscle in five ears. This audiometric finding serves as an additional objective marker of tensor tympani contraction. TT contraction should be included in the differential for cases of low frequency mixed hearing loss, particularly if there is decreased compliance on tympanometric testing, as noted by us previously [16].

## Abbreviations

dB: Decibels
Hz: Hertz
TT: Tensor tympani
